# A unifying model for multiple sclerosis

**DOI:** 10.1007/s10238-025-01666-3

**Published:** 2025-04-30

**Authors:** Daniel Jonathan Park

**Affiliations:** 1 Melbourne Bioinformatics, Faculty of Medicine, Dentistry and Health Sciences, The University of Melbourne, Melbourne, Australia; 2 Department of Biochemistry and Pharmacology, Faculty of Medicine, Dentistry and Health Sciences, The University of Melbourne, Melbourne, Australia

**Keywords:** Multiple sclerosis, Viral reactivation, EBV, HHV-6A, Cytotoxicity, Autoantibodies, Disease model, RRMS, Progressive MS

## Abstract

Multiple sclerosis (MS) is a complex neurodegenerative disorder with unresolved cause that has been the subject of intensive research. A variety of putative models have been proposed to explain the course of disease. The preeminent mechanisms are suggested to be based on autoimmunity, including via viral epitope mimicry, although difficulties with a classical autoimmunity model for MS have been described. One prior idea that incorporates consideration of viral–self-cross-reactivity is that reactivated HHV-6A virus might induce subsequent reactivation of another virus, EBV, in a relay, resulting in a cascade of downstream consequences. Here, an alternative model for MS is proposed. This posits a viral reactivation relay in which EBV reactivation in the brain precedes HHV-6A reactivation in oligodendrocytes and neurons. At this juncture, relapsing–remitting MS (RRMS) can ensue to generate characteristic lesions, dominated by outbreaks of viral reactivation and CD8+T-cell-mediated cytotoxicity and inflammation. Additionally, self-targeting antibodies can be raised to mark the onset of progressive MS in a subset of patients. This model harmonises a plethora of prior evidence from diverse fields. It is suggested that future studies should challenge this new model for MS and that it provides direction for future approaches to prevention and therapy.

## Overview

Multiple sclerosis (MS) is the most frequent demyelinating disease, affecting approximately 2.5 million people, globally. Among young adults, MS is the leading cause of permanent disability.

MS has been defined as a chronic inflammatory disease of the central nervous system (CNS), leading to large focal lesions in the white and grey matter of the brain and spinal cord [1]. Demyelination through the destruction and loss of oligodendrocytes is accompanied by variable axonal loss and is associated with strong astroglial involvement—a dense glial scar forms in long-standing lesions. All MS cases exhibit focal lesions with prominent CD8+T-cell representation [1]. However, in progressive disease, more diffuse active demyelination and neurodegeneration is also evident, associated with microglial activity and suggested to be driven by a soluble factor produced by perivascular lymphocytes or plasma cells [1–3]. There is evidence that autoantibodies, including oligoclonal IgM autoreactive antibodies, might be associated with progressive forms of MS. Potential targets include neurofilament, sperm-associated antigen 16, the iron transport scavenger protein SCARA5 and ganglioside [4–6].

The clinical course of MS varies widely. Most patients (~ 80%) present with relapsing–remitting disease (RRMS), wherein periods of acute exacerbation of symptoms alternate with periods of recovery [2]. RRMS can progress to a secondary progressive phase (SPMS)—indeed, this occurred in 50–60% of RRMS patients in the pre-treatment era. Alternatively, about 15% of MS patients present with primary progressive disease (PPMS), where symptoms continuously advance without periods of remission. PPMS typically has a later onset than RRMS, coinciding with the age range seen for SPMS.

To this author, these patterns suggest that MS is a multi-phase disorder wherein the establishment of CD8+T-cell-dominated focal lesions might be required as a precursor to the establishment of a less focused, antibody-based progressive phase. In some cases, the progressive phase might establish very soon after focal lesions have first begun to develop, and in other cases, the progressive phase may never develop. Between these extremes, a spectrum of phenotypes are possible based on the timing of the onset of autoimmunity and the nature of the autoantibody targets across MS cases.

### Reevaluating MS as an autoimmune disorder

In the literature, MS is frequently assumed to be an autoimmune disorder. Proposed models underpinning this include: immune cross-reactivity, wherein antibodies raised against viral components are also able to target host components (for example, EBV or HHV-6 [7]); or the generation of autoantibodies influenced by an altered local inflammatory environment. Cited reasons for this assumption of MS as an autoimmune disorder include: the presence of antibody oligoclonal bands (OCBs) in the cerebrospinal fluid (CSF) of MS patients [8]; the observation of immune cells, including T lymphocytes at MS lesion sites; and reports of putative autoantibodies. Indeed, a commonly used murine model to represent MS, the experimental autoimmune encephalomyelitis (EAE) model, is based solely on an induced autoimmune response to myelin components [9].

Despite observations in support of an autoimmune component to MS, the model of MS as a classical autoimmune disorder has been questioned [10, 11]. MS does not appear to be associated with other autoimmune disorders, a criterion used in the classification of such disorders [11]. Classical autoimmune diseases present with relatively homogeneous autoantibody targets. Despite substantial effort, no dominant autoantibodies that would explain a large fraction of occurrences of MS have been described. The detected characteristic OCBs in MS are relatively stable within individual cases over time but appear to vary widely across MS cases—and no convincing consensus OCB-associated target antigen has been described. Furthermore, the EAE model for demyelination based on autoimmunity to myelin sheath components results in disseminated demyelination throughout the entire CNS, quite unlike the limited focal lesions seen in MS, and RRMS especially.

Histologically, and, again, unlike the EAE model, a MS plaque is characterised by substantially elevated levels of CD8+T cells and relatively few CD4+T cells. Indeed, pathological studies indicate that the CD8+T-cell is the primary mediator of effector function in MS plaques [11]. In contrast, the EAE model appears to be mediated primarily through CD4+T-cell Th1 and Th17 functions. Oligoclonal expansion of CD8+T cells has been reported in MS brains, blood and CSF. Such selection has not been reported for CD4+T cells. The CNS does not normally express class I MHC (which presents intracellular-derived epitopes to T-cell receptors on CD8+T cells) except in vascular cells and rare meningeal cells. Consistent with a prominent role for CD8+T cells in MS, however, during inflammatory processes such as MS, class I MHC is observed in astrocytes, oligodendrocytes and neurons, including axons [12]. Damage to myelin is not considered sufficient to explain the spectrum of symptoms in MS. Nor are levels of demyelination strictly correlated to disease stage, neurologic deficits or lesion pathology. Magnetic resonance imaging in postmortem brains has shown that axonal injury is the primary event leading to clinical deficits in MS [10]. Collectively, these observations suggest that the EAE model exhibits substantially different pathogenic pathways to MS—although it would appear to phenocopy the progressive phase of MS somewhat better than the relapsing–remitting, lesion-centric phase.

It has been identified that the B-cell receptors of B lymphocytes found in MS patient CSF largely corresponded to OCB antibodies [13]. Approximately one-third of these were shown capable of binding to EBV proteins or peptides, including EBNA1. Eight of nine MS patients exhibited antibodies capable of binding EBNA1 peptides. It was demonstrated that EBNA1 epitope AA386-405, previously suggested as a candidate molecular mimic and inducer of self-targeting antibodies, can yield cross-reactivity with GlialCAM peptide (GlialCAM is expressed on oligodendrocytes). However, cross-reactivity with GlialCAM appeared to occur in a relatively small fraction of MS cases [13, 14], and the significance of this observation to MS pathology is unclear. The authors demonstrated that inoculation with EBNA1 epitope AA386-405 could exacerbate the mouse EAE model, but as discussed earlier, the EAE model does not appear to represent the biology of MS rigorously.

### Aetiology and epidemiology

A variety of factors have been associated with MS onset or progression. A seminal epidemiological study demonstrated that Epstein–Barr Virus (EBV) is necessary but not sufficient for the substantial majority of MS [15]. The age of first exposure to EBV appears to be very influential—EBV-seroconverted young children have a relatively low risk of subsequently developing MS. Conversely, EBV-associated mononucleosis after the onset of puberty is a significant risk factor [16]. It appears likely that hormonal effects on the immune system have an important role in this effect.

EBV-infected B cells are found in the brains of people with MS as well as healthy controls and have been shown to persist and reactivate in ectopic B-cell follicles that form in the meninges of MS patients [17]. Such follicles represent major reservoirs of EBV. EBV is a complex virus known to employ a diverse array of tactics to avoid eradication. As well as latency programming, EBV influences the immune system and can induce immune suppression [16–20]. Recent analysis of postmortem MS brains containing B-cell-enriched perivascular infiltrates and meningeal follicles demonstrated CNS-specific and PD-L1- and PD-1-mediated immunomodulation [17]. EBV is known to produce a viral homologue to the human immune-suppressive cytokine interleukin-10 (vIL-10) and to result in the release of exosomes containing numerous factors that could have roles in signalling and immunomodulation [19, 21]. A variety of biological factors can induce reactivation of EBV [22].

Sex is an important risk factor for MS—females are about three times more likely to develop RRMS than their male counterparts, although this imbalance is less pronounced for SPMS (~ twofold) and disappears for PPMS. Other risk factors for MS include smoking, body mass index, diet, vitamin D levels, UV exposure, a variety of infectious agents and many genetic factors [23, 24].

Prominent among the genetic factors are those that influence the immune system and particularly noteworthy is the MHCII allele DRB1*15:01, which confers a relatively large effect size. Notably, MHCII molecules function as co-receptors for EBV infection, and the “risk allele” DRB1*15:01 was shown to facilitate EBV infection relative to the “protective allele” DRB1*01:01 [25].

Modest associations between MS and reactivation of the widespread HHV-6A beta-herpesvirus have been observed—based on increased HHV-6A-specific IgG, or the presence of HHV-6A-specific IgM or viral DNA in serum or CSF—without indication of a prominent causative role [7, 26, 27]. The closely related HHV-6B virus has also been the subject of substantial similar interest but does not appear associated with MS based on similar screening approaches. OCB antibody specificity against HHV-6 has been described [28, 29], and potential myelin cross-reactive antibodies induced by an immune response to HHV-6A have been reported [30]. HHV-6A infection has also been shown to accelerate disease progression in a non-human primate EAE model system [31]. Previous authors have listed a number of plausible mechanisms by which HHV-6A might induce autoimmunity [32]. These include molecular mimicry as well as an altered inflammatory environment that is conducive to raising responses to self-epitopes that would otherwise be unlikely to occur. Notably, HHV-6A infection interacts (in an epidemiological sense) with other factors suspected of modulating MS susceptibility and progression, including EBV [33–35].

HHV-6A has tropism for a relatively broad variety of cell and tissue types, owing to use of the complement receptor CD46, expressed on all nucleated human cells, as a cellular receptor [36, 37]. Organs infected by HHV-6A include the brain, salivary glands, tonsils, liver, kidneys and lymph nodes [38]. Of particular note, HHV-6A is a neurotropic virus that enters the CNS mainly through the olfactory pathway and, once there, may infect several cell types [39]. Compellingly, among these are oligodendrocytes (responsible for producing protective myelin sheaths around the axons of neurons) and neurons [40, 41]. HHV-6A establishes a latent infection in CNS cells in vivo. However, in certain circumstances, such as immune impairment, it may reactivate so that viral particles can be detected at low levels in the CSF of patients. Similar to EBV, cytokines are believed to contribute to HHV-6 reactivation [22, 42].

Fiertz postulated a model for MS that involves HHV-6A infection causing EBV reactivation in the brain of MS patients, leading to intrathecal B-cell transformation [43]. This would result in an immune response against EBV-infected cells as a basis for inflammatory lesion formation in the brain of susceptible individuals. Fiertz further proposed that HHV-6A and EBV might induce expression of the human endogenous retrovirus HERV-K18-encoded superantigen to induce secondary local autoimmune phenomena. However, EBV cellular tropism would appear to be a significant weakness of this model—when EBV DNA or antigen is reported to be present in the CNS, it is typically found in infiltrating inflammatory cells, including B cells and plasma cells rather than oligodendrocytes or neurons [14, 44, 45].

Conversely, reactivation of HHV-6A by EBV has not been studied but appears plausible. We know that EBV can influence the immune system in diverse ways, including the creation of an immunocompromised state. As noted earlier, it is also known that HHV-6A is frequently reactivated in immunocompromised patients, including those affected by DRESS (drug reaction with eosinophilia and systemic symptoms), AIDS patients and bone marrow transplant recipients [27, 46]. EBV reactivation is also known to alter the cell signalling/cytokine environment—for example, replication of the virus HCV has been shown to be boosted by vIL-10 [22].

### HHV-6A reactivation and MS associations have likely been underestimated

There are numerous reasons to suspect that prior association studies might have markedly under-represented the relevance of HHV-6A reactivation to MS. Testing for HHV-6 infection can be highly nuanced due to challenges in differentiating HHV-6A from HHV-6B, temporal effects, local isolation and low viral copy number [46]. In terms of false-positive effects, since HHV-6A has relatively wide cell and tissue tropism, it is likely that viral reactivations can occur in cells and tissues that have no influence on MS pathogenesis, particularly if we assume MS lesion-local infection to be important. Such remote reactivations could result in increased levels of virus-specific IgM, IgG or DNA in tissues without relevance to MS pathology and could contribute HHV-6A signal in sampled tissue (e.g. serum and PBMCs) in the absence of MS lesion-relevant reactivation. Regarding false-negative effects, aspects of the viral life cycle and reactivation detection methods are problematic. HHV-6A can reactivate in local regions of tissues transiently and without the generation of high viral DNA copy number, rendering it difficult to detect systemically [47, 48]. HHV-6A reactivations appear to emerge in relatively short bursts prior to resumption of latency [49]. Serial serum PCR-based testing in bone marrow transplant recipients indicated that HHV-6A reactivation is a frequent phenomenon in this immunocompromised state, albeit within narrow time windows [48]. HHV-6 spreads largely via cell-to-cell contact and possibly via exosomes [21, 50, 51]. Such infectious modes do not require lytic release or high viral titres.

Additional complexity around HHV-6A-MS association studies relates to the resolution of HHV-6A versus HHV-6B. Many studies were not designed to distinguish these two highly similar viruses, although latter PCR-based studies increasingly have. The vast majority of serological studies have not differentiated HHV-6A and HHV-6B. Relatively recent work has attempted to address this but apparently at the expense of sensitivity [35, 52].

Together, these indicate that HHV-6A has the potential to play a significantly larger role in MS pathology than molecular epidemiological screening methods have been capable of resolving thus far. While less compelling based on current evidence, it also remains possible that the broad systemic tropism of HHV-6B has clouded the picture regarding a causative role for HHV-6B in the pathogenesis of MS.

### A unifying model—EBV induces HHV-6A reactivation

Here, a core model for MS is proposed. In this model, EBV-infected B cells migrate to the brain to establish long-term latent colonisation. Additionally, pockets of CNS oligodendrocytes and/or neurons become latently infected with HHV-6A. After these persistent infections have been established, EBV within brain-resident B cells can be reactivated by diverse triggers, influenced by the underlying host immune status. Reactivated EBV then facilitates the reactivation of HHV-6A in oligodendrocytes and/or neurons. This stimulus for HHV-6A reactivation could comprise direct signalling by secreted factors and/or induction of immunosuppression. Reactivated HHV-6A could induce demyelination and axonal damage via a number of mechanisms. Persistent infection and reactivated HHV-6A and CD8+T-cell-mediated cytotoxic responses are proposed to be the basis for focal lesion formation, wherein rounds of viral reactivation reflect the relapsing–remitting pattern seen in RRMS. After the formation of local lesions, progressive MS can result from the generation of autoantibodies via a number of mechanisms proposed previously in the literature [32].

### Observations consistent with this model

This model appears consistent with many observations reported in the literature, which can be grouped for convenience by the categories: EBV and associated biology; HHV-6A and biology; pathology and therapeutic responses.

Regarding EBV, this virus appears to be required for the onset of MS. Furthermore, DRB1*15:01, an effective viral entry receptor for EBV, represents a major MS risk allele.

The wide range of known risk factors for MS converge on immune system influences that are relevant to EBV infection patterns and reactivation propensity. Oligoclonal antibodies in MS do not appear to dominantly exhibit specific self-targeting properties—rather they could predominantly represent “innocent bystander” products from ectopic brain-localised EBV-infected B-cell-derived populations combined with, to varying degrees, antibodies raised against EBV and HHV-6A as a result of reactivations. B cells producing antibodies that target EBV epitopes have been shown to occur in the CSF of MS cases with apparently variable levels of involvement across patients [13, 53]. Reactivated EBV is known to induce altered immune states, signalling by cytokines and exosomes, and the reactivation of other viruses.

With respect to HHV-6A, HHV-6A associations with MS have been observed, but due to viral life cycle and mode of transmission, a wide tropism and the transient nature of systemic detectability, the likelihood is that reported associations represent a gross underestimate. HHV-6A is known to infect oligodendrocytes and neurons in vivo and has been detected at the site of MS lesions. Anti-HHV-6 antibodies have been described in the CSF and OCBs of MS patients. HHV-6A is known to reactivate in immunocompromised states.

In terms of pathology, the limited number of focal lesions in RRMS and the more diffuse profile of damage during progressive disease, that is associated with a soluble component and microglial involvement, aligns with: reactivating viral pockets and CD8+cytotoxicity having a dominant effect in RRMS; and a switch to autoantibodies driving the progressive phase of the disease. A high CD8+:CD4+T-cell ratio and clonal enrichment for CD8+, but not CD4+T cells, is typical in MS lesions. Regarding an autoimmune component, no consensus autoantibody targets have been demonstrated for a large proportion of MS cases. Possibly, this is in part due to autoantibodies not driving RRMS in the absence of the progressive phase of the disease. Further, there is potential for numerous viral epitopes to induce self-cross-reactivity. Alternatively, and perhaps co-occurring with other modes of autoantibody generation, the altered immune environment of viral-induced neuroinflammation could yield a variety of potential self-targets without viral mimicry.

Concerning MS therapeutic responses, B-cell ablation therapy is a relatively effective treatment for MS. This would be expected to target EBV-infected B cells involved in a viral reactivation relay as well as the production of autoantibodies during progressive disease. Variable responses to plasma exchange therapy according to MS subtypes fit with the concept that autoantibodies have varying influence across MS patients, depending on whether progressive disease onset has occurred. Plasma exchange therapy has varying clinical effectiveness across RRMS and progressive forms of MS and is a second-line treatment for RRMS but not an approved treatment for progressive MS. It is not curative, however, merely offering temporary relief from symptoms in some cases [54–56]. It is important to note that plasma exchange therapy removes soluble components from blood that include diverse factors such as cytokines and immune complexes and not only antibodies. It is possible that plasma exchange may influence viral reactivation or immune cell signalling during RRMS. That plasma exchange is not a clearly effective therapy for progressive MS remains consistent with the subject proposed model, since immune cells involved in generating autoantibodies would remain and, further, brain-local factors may not be effectively removed by this therapy.

### Future directions

While the presented model appears to resolve many difficulties with prior hypotheses and harmonise a broad variety of evidence modes, it does rely on the notion that HHV-6A reactivation detection at active MS lesions has been inaccurately assigned in prior studies. It also does not preclude the idea that other viral types could substitute the role of HHV-6A in the second stage of the viral reactivation relay. For example, it could be argued that HHV-6B reactivation might have been similarly mis-assigned in the previous studies—perhaps in more exaggerated fashion owing to different cellular and tissue tropisms. Other agents, such as human endogenous retroviruses [57], have also received interest and warrant further investigation.

Future studies should focus on the potential for reactivation of HHV-6A induced by reactivated EBV and the underlying mechanisms. The true fraction of MS sufferers infected with HHV-6A is unknown and, the challenges associated with accurate detection notwithstanding, efforts to establish this should be prioritised. It would be useful to conduct postmortem screening of MS lesions and appropriate control tissues with new sncRNA FISH probes. In a similar vein, it would be helpful if non-probe-based, sensitive assays for the detection of sncRNA markers of HHV-6A reactivation were developed. These might be applied to serially sampled CSF and compared with relevant control samples. The proposed model is also partially testable in terms of the distinction between non-progressive and progressive diseases. Under the proposed model, we would expect a higher relative frequency of autoantibodies to myelin and neural-specific targets in the latter setting. Pursuing this line of enquiry might make use of single B-cell resolution analysis and subsequent screening [13].

The model provided here, along with prior arguments made by others, calls into question the validity of the mouse EAE model, which does not involve EBV in the disease process nor involve focal lesions with CD8+T-cell prominence. Inferences made following experiments that have made use of such models must be treated with caution and better experimental systems sought.

If the model presented in this article—of reactivating EBV relaying to reactivate HHV-6A and induce MS pathology—stands up to future scrutiny, it could provide the foundation for more focused therapeutic interventions, including vaccination strategies and specific antivirals [58]. Evidence has been presented to indicate that the antiviral agent acyclovir might decrease the frequency of MS relapses [59]. Perhaps more potent and specific EBV and/or HHV-6A antivirals could provide more pronounced clinical benefits. Given that infectious mononucleosis caused by EBV is a major risk factor for MS, early childhood vaccination against EBV might reduce the risk of later viral reactivation in brain-ectopic B-cell colonies. Early childhood exposure to competent EBV apparently provides protection against MS, although this benefit should be weighed seriously against adverse health outcomes resulting from later exposure. Similarly, the risk of HHV-6A pockets of infection in the brain might be limited by appropriate vaccination strategies. Therapeutics that target EBV signalling, such as vIL-10 or interventions that lower risks of EBV reactivations, might also prove effective in preventing or treating MS.

## Conclusion

Here, a harmonising model for MS is presented. This viral reactivation relay model (EBV-HHV-6A) considers local lesions that are driven by CD8+T-cell cytotoxic effects on reactivated HHV-6A-infected oligodendrocytes and neurons, reflecting RRMS. It also proposes a switch to progressive MS driven by the development of self-directed antibodies that result from reactivated HHV-6A. The model aligns well with a large body of prior evidence from diverse fields and paves the way for future experimental design and clinical interventions.

## Data Availability

Not applicable.

## Abbreviations

CNS: Central nervous system
CSF: Cerebrospinal fluid
EAE: Experimental autoimmune encephalomyelitis
EBNA1: Epstein–Barr nuclear antigen 1
EBV: Epstein–Barr virus
HCV: Hepatitis C virus
HHV-6A: Human herpesvirus-6A
MHC: Major histocompatibility complex
MS: Multiple sclerosis
OCB: Oligoclonal band
PPMS: Primary progressive multiple sclerosis
RRMS: Relapsing–remitting multiple sclerosis
SPMS: Secondary progressive multiple sclerosis
vIL-10: Viral interleukin-10
